# Case series analysis of immunophenotypic evolution during recurrence of low-grade endometrial stromal sarcoma: implications for molecular stability and clinical management

**DOI:** 10.3389/fonc.2026.1823340

**Published:** 2026-06-02

**Authors:** Jing Li, Lihong Zhang, Tingting Chen

**Affiliations:** Obstetrics & Gynecology Hospital of Fudan University, Shanghai Key Lab of Reproduction and Development, Shanghai Key Lab of Female Reproductive Endocrine Related Diseases, Shanghai, China

**Keywords:** case series, immunophenotypic evolution, JAZF1, low-grade endometrial stromal sarcoma, PHF1, recurrence

## Abstract

**Purpose:**

To investigate the pathological morphology, immunophenotype, and molecular genetic characteristics of recurrent low-grade endometrial stromal sarcoma (LG-ESS) through the analysis of paired primary and recurrent tumor samples.

**Methods:**

A single-center retrospective analysis was conducted using the clinical data, pathological morphology, immunohistochemical results of estrogen receptor, progesterone receptor (PR), CD10, Ki-67, and fluorescence *in situ* hybridization findings for *JAZF1* and *PHF1* gene rearrangements in paired tumor tissues from four patients with LG-ESS.

**Results:**

Patients’ ages ranged from 22 to 34 years. All cases showed consistent *JAZF1* or *PHF1* gene rearrangements in both primary and recurrent tumors. However, immunophenotypic alterations were observed during recurrence: one recurrent lesion showed loss of PR expression accompanied by a decreased Ki-67 index, whereas another demonstrated loss of CD10 expression. The median follow-up time was 84 months (range, 36–144 months). The recurrence interval ranged from 3 to 12 years, and all patients remained alive following comprehensive treatment.

**Conclusion:**

This case series indicates that core driver gene rearrangements remain stable during recurrence in LG-ESS, whereas immunophenotypic evolution may occur in some cases. These preliminary findings suggest that the re-evaluation of biomarkers such as hormone receptors at recurrence may provide valuable guidance for individualized treatment selection (e.g., hormonal therapy). Given the characteristic tendency for late recurrence in LG-ESS, long-term follow-up is essential.

## 1 Introduction

Low-grade endometrial stromal sarcoma (LG-ESS) is a rare uterine mesenchymal tumor that predominantly affects young women. Standard treatment includes surgery, chemotherapy, and hormonal therapy (1). A significant clinical characteristic is its strong propensity for recurrence, which can occur many years after initial treatment (2, 3). Such recurrence imposes substantial long-term physical and psychological burdens on patients. Molecularly, characteristic gene fusions such as *JAZF1*-*SUZ12* are highly stable in LG-ESS and serve as important diagnostic markers (4). Clinically, positivity for hormone receptors (ER/PR) makes hormonal therapy an important adjuvant or systemic treatment strategy (3, 5). However, clinical observations indicate that some recurrent tumors develop resistance to previously effective hormonal therapies although the underlying mechanisms remain unclear (1).

Recurrence of LG-ESS is primarily diagnosed through the detection of new tumor lesions on postoperative imaging (6, 7). Typical manifestations include nodules in the endometrial region, myometrial invasion, or extension along vessels and ligaments, and may involve distant metastases in the abdominal cavity and lungs (8). LG-ESS exhibits indolent growth with late and potentially repeated recurrences (9). Recurrence can be further confirmed through combined evaluation of pathological morphology with positive estrogen receptor (ER)/progesterone receptor (PR) expression (10).

Currently, longitudinal studies investigating the recurrence mechanisms in LG-ESS remain limited, particularly regarding systematic analyses of immunophenotypic evolution in paired samples. Whether such evolution is associated with treatment resistance remains an important unresolved clinical question (11, 12). Through longitudinal analysis of four paired primary and recurrent LG-ESS samples, this study aimed to systematically characterize the dynamic evolution of immunophenotypes during recurrence and explore focus their potential association with tumor clonal evolution and clinical treatment, particularly hormonal therapy response). The findings may provide new insights into the individualized management of recurrent LG-ESS.

## 2 Materials and methods

### 2.1 Case selection

We retrospectively collected four LG-ESS cases with paired primary and recurrent tumor tissues diagnosed in the Department of Pathology, Obstetrics and Gynecology Hospital of Fudan University, between 2009 and 2025.

Inclusion criteria were as follows: (1) diagnosis confirmed independently by two senior pathologists; (2) complete clinical and pathological data; and (3) sufficient paraffin-embedded tissue available for subsequent analyses. Cases with concurrent malignancies or insufficient tissue samples were excluded.

#### 2.1.1 Data collection and variables

For each included patient, the following variables were systematically collected from medical records and pathological archives:

Demographic and clinical data: age at diagnosis, clinical presentation (e.g., abnormal bleeding), surgical procedure, adjuvant therapy (type and duration), and recurrence data.

Pathological data (for both primary and recurrent samples):

Histological features: tumor cell morphology, mitotic count per 10 high-power fields (HPF), presence of necrosis, vascular invasion, and smooth muscle, fibrous, or sex cord-like differentiation.
Immunohistochemical markers: expression status of ER, PR, CD10, and Ki-67 proliferation index.
Molecular genetic status: *JAZF1* rearrangement status detected via fluorescence *in situ* hybridization (FISH) using break-apart probes.

#### 2.1.2 Pathological evaluation

Two pathologists independently reviewed the samples in a double-blinded manner. Evaluations included tumor cell morphology, mitotic count per 10 (HPF), necrosis, vascular invasion, and areas with smooth muscle, fibrous, or sex cord-like differentiation.

#### 2.1.3 Tumor staging

Clinical staging was performed according to the American Joint Committee on Cancer (AJCC) 8th edition TNM classification, harmonized with the 2015 International Federation of Gynecology and Obstetrics (FIGO) staging system for uterine sarcomas (13, 14).

### 2.2 Immunohistochemical staining

Immunohistochemistry (IHC) for ER, PR, CD10, and Ki-67 was performed on 4-µm thick formalin-fixed, paraffin-embedded tissue sections using a standard automated protocol on a Leica Bond fully automated stainer, with appropriate positive and negative controls. Antigen retrieval and antibody dilution conditions were optimized through preliminary experiments (e.g., ER/PR antibody clones 6F11 and 16 at a working dilution of 1:100). Two pathologists independently evaluated the staining results.

IHC expression was assessed by quantifying the overall proportion of positive tumor cells (0–100%). Specimens were categorized as negative (no detectable staining or <1% positive cells), 1+ (1%–25% positive), 2+ (26%–50% positive), or 3+ (>50% positive) (11). Additionally, samples were evaluated using the H−score method (15, 16), which integrates both the proportion of immunoreactive cells and intensity of staining (1+ = weak, 2+ = moderate, and 3+ = strong). The final H-score (0–300) was calculated using the formula: (1 × % of cells 1+) + (2 × % of cells 2+) + (3 × % of cells 3+). A threshold of ≥1% positive cells was applied to dichotomize cases as positive or negative. The two pathologists also independently confirmed the concordant interpretation.

### 2.3 Fluorescence *in situ* hybridization

FISH was performed on paired paraffin sections using *JAZF1* and *PHF1* break-apart probes. The procedure included dewaxing, protease digestion, co-denaturation of probe and target DNA, hybridization, and washing. A positive rearrangement was defined as >15% of cells exhibiting split red/green signals in at least 100 intact nuclei.

### 2.4 Ethics approval

All experiments were conducted in accordance with protocols approved by the Ethics Committee of the Obstetrics & Gynecology Hospital of Fudan University (approval number: 2025-275). All patient samples were collected and processed according to institutional ethical and safety regulations. The study adhered to the ethical principles of the Declaration of Helsinki. Written informed consent was obtained from all patients after detailed explanation of the study’s purpose, specimen collection procedures, data usage, and potential risks.

### 2.5 Data collection and analysis

All clinical and pathological data were retrospectively collected. The interval to recurrence was defined as the time (months) from initial definitive surgery to the first pathologically confirmed recurrence (17). This interval is presented descriptively in the results tables.

Categorical variables were compared using the chi-square (χ²) test or Fisher’s exact test, as appropriate. All statistical analyses were performed using SPSS 26.0 (IBM Corp.), and a two-sided p-value <0.05 was considered statistically significant.

## 3 Results

### 3.1 Clinical and pathological characteristics

**Figure 1:**
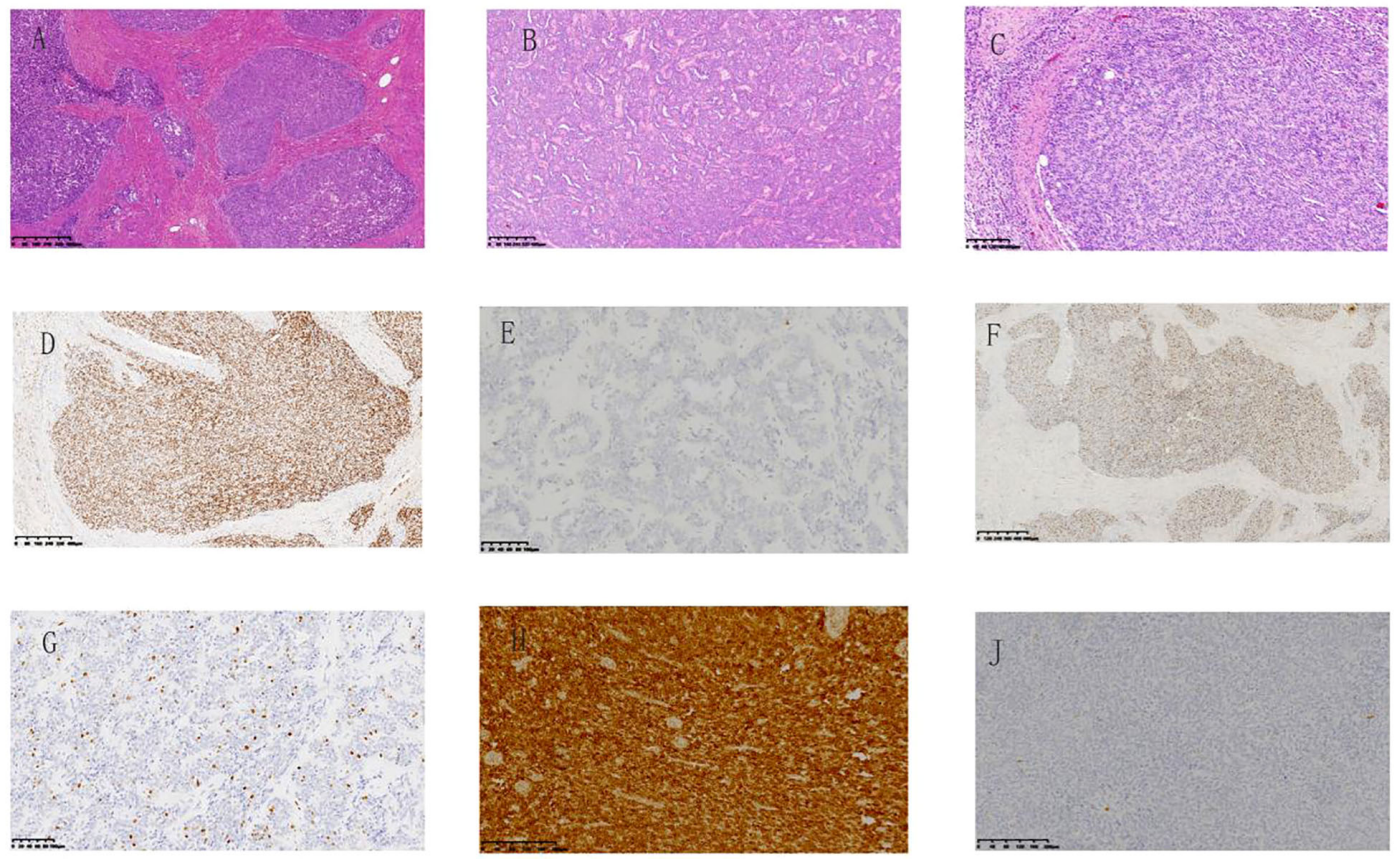
Morphological and immunohistochemical changes between primary and recurrent low-grade endometrial stromal. **(A–C)** Comparison of histomorphological features between the primary and recurrent low-grade. The primary tumor showed an infiltrative growth pattern **(A)**, whereas the recurrent tumor exhibited papillary and sex cord-like differentiation **(B, C)**. **(D–J)** Immunohistochemical phenotypic changes. Case 1 demonstrated loss of PR expression **(D, E)**, with a decreased Ki67 proliferation index **(F, G)**. Case 2 demonstrated loss of CD10 expression **(I, J)**.

The patients were aged 22–34 years and presented with abnormal vaginal bleeding or pelvic masses (**Table 1**). Initial treatment in all cases included total hysterectomy, with ovarian preservation in two cases. Notably, Cases 1 and 2 demonstrated extrauterine dissemination at recurrence. Case 2 developed lung metastasis, a well-documented metastatic site for LG-ESS (18), thereby confirming the metastatic potential of this tumor. In all cases, recurrence was confirmed histopathologically using biopsy or surgical specimens following initial clinical and/or imaging suspicion. This multimodal assessment approach, integrating clinical symptoms, imaging findings, and pathological confirmation, is consistent with standard diagnostic practice for recurrent LG-ESS (10). All primary tumors exhibited classic LG-ESS morphology, including tongue-like myometrial infiltration (**Figure 1A**). The recurrent tumors demonstrated certain morphological alterations. Case 1 developed papillary structures (**Figure 1B**) and sex cord-like differentiation (**Figure 1C**), accompanied by a reduced mitotic count. Case 2 showed mildly increased cellular atypia.

**Table 1:** Clinical characteristics and treatment outcomes of patients.

Case	Age	Surgical approach	Extrauterine extension	Tumor thrombus	LN metastasis	Stage	Initial treatment	DFS (M)	Site of recurrence	Post-recurrence treatment	Follow-up status	Outcome
1	30	TH + BSO	No	Yes	No	I	Chemotherapy	144	Abdominal wall, intestinal tract, and pelvis	Chemotherapy	Regular follow-up	Alive
2	22	TH + BSO + pelvic lymphadenectomy + parametrial lesion resection	Yes	Yes	No	II	Chemotherapy	120	Right iliac vessel region and right lung	Chemotherapy	Regular follow-up	Alive
3	34	Single-port laparoscopic resection of retroperitoneal mass	No	No	No	II	Letrozole 2.5 mg oral, once daily	36	Lateral peritoneum	Chemotherapy	Regular follow-up	Alive
4	28	TH + bilateral fallopian tubes	No	No	No	I	None	60	Multiple pelvic nodules	Chemotherapy	Regular follow-up	Alive

LN, lymph node; DFS, disease−free survival; TH, total hysterectomy; BSO, bilateral salpingo-oophorectomy; M, month.

DFS was defined as the time from initial surgery to first pathologically confirmed recurrence (in months).

### 3.2 Immunohistochemical phenotypic evolution

Immunohistochemical results revealed phenotypic alterations in some recurrent tumors (**Table 2**). Case 1: The recurrent tumor completely lost PR expression (**Figure 1D**: primary tumor strongly positive; **Figure 1E**: recurrent tumor negative), whereas the Ki-67 index significantly decreased from 40% to 5% (**Figures 1F, G**). ER and CD10 expression were retained. Case 2: The recurrent tumor lost CD10 expression (**Figure 1H**: primary tumor positive; **Figure 1I**: recurrent tumor negative), whereas ER, PR, and Ki-67 remained stable. Cases 3 and 4: All markers showed consistent expression patterns between primary and recurrent tumors. Overall, two of the four paired samples (50%) exhibited definitive immunophenotypic alterations at recurrence, including one case with PR loss accompanied by reduced Ki-67 expression and one case with CD10 loss. ER/PR expression remained stable in the remaining cases, reflecting heterogeneity in tumor clonal evolution (11, 12).

**Table 2:** Comparison of immunohistochemical markers between primary and recurrent tumors.

Case	Marker	Primary tumor	Recurrent tumor	Concordant
1	ER	Positive	Positive	Yes
	PR	Positive	Lost	No
	CD10	Positive	Positive	Yes
	Ki-67	40%	5%	No
2	ER	Positive	Positive	Yes
	PR	Positive	Positive	Yes
	CD10	Positive	Lost	No
	Ki-67	20%	20%	Yes
3	ER	Positive	Positive	Yes
	PR	Positive	Positive	Yes
	CD10	Positive	Positive	Yes
	Ki-67	5%	5%	Yes
4	ER	Positive	Positive	Yes
	PR	Positive	Positive	Yes
	CD10	Diffusely positive	Positive	Yes
	Ki-67	5%	5%	Yes

ER, estrogen receptor; PR, progesterone receptor; CD10, cluster of differentiation 10; Ki-67, proliferation index.

### 3.3 Molecular genetic characteristics

**Figure 2:**
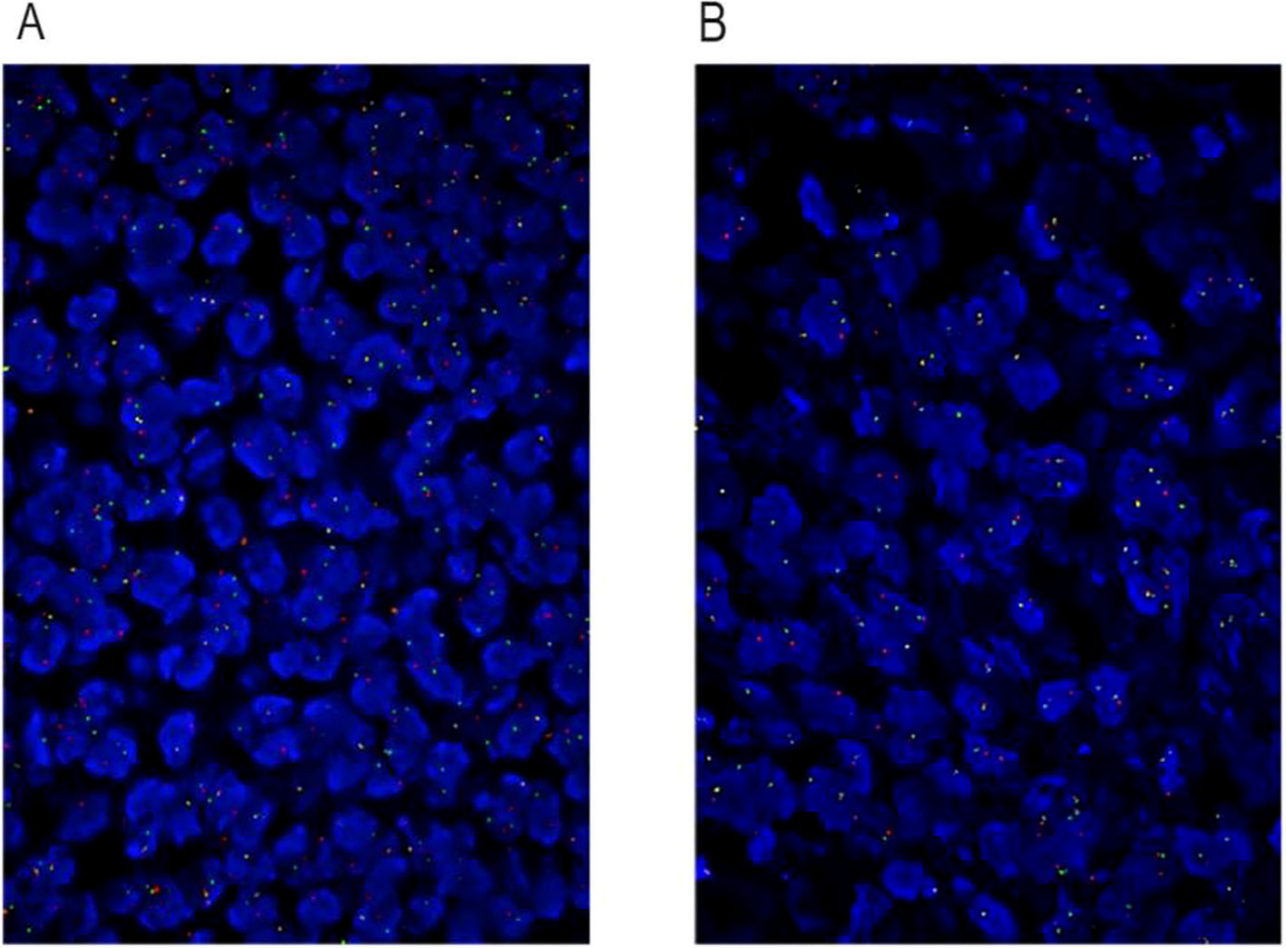
FISH findings in low-grade endometrial stromal sarcoma. **(A, B)** Identical FISH findings were observed between primary and recurrent tumors, including JAZF1 **(A)** and PHF1 **(B)**.

FISH analysis (**Figure 2**) revealed that all four patients had completely identical gene rearrangement patterns between the primary and recurrent tumors (**Table 3**). Cases 1–3 harbored *JAZF1* rearrangements, whereas Case 4 exhibited a *PHF1* rearrangement. These findings strongly support the clonal homology between recurrent tumors and their corresponding primary tumors and indicate significant stability of driver gene fusions during disease progression (12, 19).

**Table 3:** Molecular genetic features of primary and recurrent low-grade endometrial stromal sarcoma detected via FISH.

Case No.	Primary tumor FISH result	Recurrent tumor FISH result	Molecular concordance
1	*JAZF1* Translocation	*JAZF1* Translocation	Concordant
2	*JAZF1* Translocation	*JAZF1* Translocation	Concordant
3	*JAZF1* Translocation	*JAZF1* Translocation	Concordant
4	*PHF1* Translocation	*PHF1* Translocation	Concordant
Total	Positive: 4/4(100%)	Positive: 4/4(100%)	Concordance: 8/8(100%)

FISH, fluorescence *in situ* hybridization; *JAZF1*, Juxtaposed with Another Zinc Finger Protein 1; *PHF1*, PHD Finger Protein 1.

### 3.4 Treatment and follow-up

The median follow-up duration was 84 months (36–144 months). All patients experienced recurrence, at an interval of 3–12 years, highlighting the characteristic tendency for late recurrence in LG-ESS (4, 10). Post-recurrence treatment modalities varied and included repeat cytoreductive surgery, chemotherapy, and hormonal therapy such as letrozole (18). Despite undergoing multiple lines of treatment and experiencing repeated recurrences, all patients were alive at the last follow-up, underscoring the prolonged and complex clinical course of advanced LG-ESS (**Table 1**) (4).

## 4 Discussion

This study has several strengths. First, to the best of our knowledge, it represents one of the few longitudinal paired analyses of primary and recurrent LG-ESS, enabling the direct observation of immunophenotypic evolution (e.g., PR loss and decreased Ki-67 expression) against a stable molecular background defined by *JAZF1/PHF1* rearrangements. Second, the identification of discordant hormone receptor status at recurrence raises a clinically actionable question with direct implications for treatment planning. Third, despite the limited sample size, this study systematically documents key behavioral characteristics of LG-ESS, including late recurrence and metastatic potential, which are often under-reported in the literature. Finally, the long follow-up duration, extending up to 12 years, provides valuable insights into late recurrence patterns.

Through a longitudinal analysis of four paired samples, this study provides preliminary evidence that recurrent LG-ESS demonstrates dynamic immunophenotypic evolution superimposed on a stable molecular framework characterized by *JAZF1/PHF1* rearrangements.

The observed molecular stability is consistent with findings from previous studies (4), further supporting fusion genes such as *JAZF1-SUZ12* as diagnostic “gold standard” markers for LG-ESS. FISH detection of these fusions maintains high diagnostic value even in morphologically atypical, recurrent, or metastatic lesions (4, 19).

Comparison with previous studies revealed that PR loss was identified in one of four cases (25%), consistent with reported hormone receptor discordance in recurrent LG-ESS, a phenomenon attributed to tumor clonal evolution (11, 12). Previous studies have reported PR loss in 45% of recurrent LG-ESS cases compared with matched primary tumors, suggesting that hormonal phenotype evolution is not uncommon. Similarly, CD10 loss, observed in one case in the present study, has also been noted in dedifferentiated or morphologically transformed LG-ESS (20), although its frequency remains poorly characterized in unselected cohorts.

Regarding molecular stability, the complete concordance of *JAZF1/PHF1* rearrangements between paired samples observed in this study is also consistent with previous reports (19).

Collectively, these findings contribute to the growing evidence that, although the molecular backbone of LG-ESS remains stable, the immunophenotype—particularly hormone receptor expression—may evolve and potentially influence clinical management (11, 12, 17, 19). The variability of the immunophenotype and its clinical implications constitute particularly noteworthy findings in this case series. In Case 1, the loss of PR expression accompanied by decreased Ki-67 expression in the recurrent tumor may suggest the evolution of a tumor subclone characterized by slower proliferation and reduced hormone dependence (11, 12). This finding is clinically important because hormonal therapy, including progestins and aromatase inhibitors, remains a cornerstone of systemic treatment for LG-ESS (4). Retrospective studies have indicated that the hormone receptor status in recurrent tumors may influence prognosis and therapeutic response (17). Although the small sample size precludes definitive conclusions regarding the efficacy of subsequent hormonal therapy following PR loss, the findings raise an important clinical question: whether ER/PR status should be routinely re-evaluated in recurrent LG-ESS to guide hormonal treatment selection (17).

The major clinical implication of this study is the potential value of re-evaluating biomarkers such as ER and PR when tissue from recurrent tumors is available for pathological confirmation. Such re-evaluation may provide important guidance for individualized systemic treatment strategies, including selection of hormonal drugs (3, 17). However, the generalizability and definitive clinical value of this exploratory observation require further validation in larger prospective multicenter studies.

The findings also reaffirm several established clinical and behavioral characteristics of LG-ESS. First, the longest recurrence interval reached 12 years, emphasizing the necessity of lifelong follow-up (2, 4). Second, the occurrence of distant metastasis in this cohort is consistent with the known invasive behavior of LG-ESS, including reported invasion into the vena cava or even the heart (9, 21, 22). Third, although all patients survived long-term, each experienced multiple recurrences, highlighting the limitations of current chemotherapy, radiotherapy (4), and hormonal therapy in controlling advanced disease.

The major limitation of this study is the small sample size (2), reflecting the rarity of LG-ESS. Consequently, the prevalence and clinical significance of immunophenotypic evolution require confirmation in larger prospective multicenter studies. Additional limitations should also be acknowledged. First, the retrospective design introduces potential selection and information biases, as not all recurrent cases underwent biopsy, and treatment heterogeneity may have confounded the interpretation of the observed immunophenotypic changes. Second, this was a single-center experience, which limits the generalizability of the findings to other institutions and patient populations. Future multicenter prospective studies with standardized biopsy and treatment protocols are needed to validate the prevalence and clinical impact of immunophenotypic evolution in recurrent LG-ESS. Furthermore, the lack of deeper omics analyses, such as transcriptomic or whole-exome sequencing, precluded the investigation of downstream molecular pathways potentially driving phenotypic evolution, including pathways involving biomarkers such as *FGF12* (23).

### 4.1 Summary of clinical and pathological findings

First, the disease demonstrated a propensity for late recurrence (36–144 months), including recurrence intervals extending up to more than decades (4, 7), as well as distant metastasis, consistent with its indolent yet persistent and potentially aggressive behavior (9, 22). Second, complete stability of the defining *JAZF1/PHF1* rearrangements was observed across all paired samples, underscoring their role as clonal diagnostic markers even at recurrence (12). Third, and most notably from a translational perspective, immunophenotypic evolution was identified in a subset of cases, including the loss of PR and CD10 expression. These findings suggest that hormone receptor status at recurrence may differ from that of the primary tumor, as previously reported (11, 12), thereby supporting the clinical practice of rebiopsy in recurrent tumors to guide subsequent therapy (17). Collectively, these findings reinforce the dual nature of LG-ESS: a stable genomic backbone accompanied by a dynamic phenotypic landscape that can inform personalized management strategies.

### 4.2 Implications for clinical management

Based on these findings, the following clinical recommendations are proposed:

Long-term surveillance strategy: Clinicians should inform patients regarding the risk of late recurrence, including beyond 12 years, and implement follow-up strategies extending beyond 10 years, prioritizing clinical examination and imaging surveillance.
Biopsy at recurrence: Whenever feasible, recurrent tumors should undergo biopsy for pathological evaluation. This approach serves dual purposes: confirmation of recurrent LG-ESS and re-assessment of ER/PR status to guide subsequent hormonal therapy selection, such as progestins versus aromatase inhibitors.
Ovarian preservation counseling: In premenopausal patients, the decision regarding ovarian preservation during initial surgery should be carefully balanced against the potential increased risk of recurrence, and patients should be appropriately counseled regarding this uncertainty.

### 4.3 Conclusion

This case series demonstrates that recurrent LG-ESS exhibits a characteristic pattern of a “stable molecular framework with plastic immunophenotype.” Core gene fusions serve as stable clonal markers, whereas the expression of proteins such as hormone receptors can undergo clonal evolution during recurrence. These findings have important clinical implications, suggesting that reassessing tumor biomarkers, particularly hormone receptor status, at recurrence may facilitate the development of more personalized treatment strategies. Given the indolent clinical course of LG-ESS, further studies are needed to elucidate the mechanisms underlying recurrence and drug resistance and to explore precision treatment strategies based on molecular and immunophenotypic profiles.

## Data Availability

The original contributions presented in the study are included in the article/supplementary material. Further inquiries can be directed to the corresponding author.
